# Bucky Tubes Induce Oxidative Stress Mediated Cell Death in Human Lung Cells

**DOI:** 10.1155/2015/560768

**Published:** 2015-05-18

**Authors:** Jaya Singhal, Surinder P. Singh, Stalin Karuppiah, Alok K. Pandey

**Affiliations:** ^1^Nanomaterial Toxicology Laboratory, CSIR, Indian Institute of Toxicology Research, P. O. Box 80, Mahatma Gandhi Marg, Lucknow 226001, India; ^2^CSIR, National Physical Laboratory, Dr. K. S. Krishnan Marg, New Delhi 110012, India; ^3^Academy of Scientific and Innovative Research, New Delhi, India

## Abstract

Unique physicochemical properties of carbon nanomaterials (CNMs) have opened a new era for therapeutics and diagnosis (known as theranostics) of various diseases. This exponential increase in application makes them important for toxicology studies. The present study was aimed at exploring the toxic potential of one of the CNMs, that is, bucky tubes (BTs), in human lung adenocarcinoma (A549) cell line. BTs were characterised by electron microscopy (TEM), dynamic light scattering (DLS), Fourier transform spectroscopy (FTIR), and X-ray diffraction (XRD). Flow cytometric study showed a concentration and time dependent increase in intracellular internalization as well as reduction in cell viability upon exposure to BTs. However, a significant increase in intracellular reactive oxygen species (ROS) production was observed as evident by increased fluorescence intensity of 2′,7′-dichlorofluorescein (DCF). BTs induced oxidative stress in cells as evident by depletion in glutathione with concomitant increase in lipid peroxidation with increasing concentrations. A significant increase in micronucleus formation and apoptotic cell population and loss of mitochondrial membrane potential (MMP) as compared to control were observed. Moreover, in the present study, BTs were found to be mild toxic and it is encouraging to conclude that BTs having outer diameter in the range of 7–12 nm and length 0.5–10 *μ*m can be used for theranostics.

## 1. Introduction

Nanotechnology has developed tremendously in past few decades which lead to the discovery and production of various nanomaterials (NMs), that is, metallic (silver and gold) and metallic oxide nanoparticles (ZnO and TiO_2_), quantum dots and carbon nanomaterials. These NMs find potential applications in diverse areas including physical, chemical, and biological sciences such as cosmetics, food packaging industries, electronics, medicines, and biomedical engineering [1–3]. In spite of wide applications, these NMs have critically drawn concerns towards their potential adverse effects to human beings.

Carbon nanotubes (CNTs) are the distinct form of carbon based nanomaterials (CNMs) comprised of single or concentrically stacked graphene sheets rolled seamlessly, showing astonishing structural and physicochemical properties. These have been explored for diverse applications ranging from electronics to biomedical applications in applied sciences such as nanoinjectors, tissue engineering, drug delivery, gene therapy, and biosensor technology [4–8]. The aforementioned exponential increase in applications of CNTs raised concerns towards the occupational threats on exposure of CNTs which has been discussed in several reports [9].

An occupational survey on exposure of engineered nanoparticles suggested that the workers got chronic obstruction and pulmonary and cardiac diseases due to exposure of nanoparticles [10]. It can be said that on exposure nanoparticles may be accumulated in different organs upon systemic circulation and will end up as waste; further their subsequent degradation may liberate particles into the environment where they may remain or circulate in food web [11, 12]. Previous studies dealing with both* in vitro* and* in vivo* cellular targets showed the toxic effects of MWCNTs on aquatic lives and some other species [13, 14]. The consumption of nanoparticles follows the demand and supply rule which ultimately pose a risk on environment as well as on society and become major concern [15].

According to previous reports, MWCNTs were found to be immunotoxic when exposed to murine macrophages for 16 h, 24 h, and 32 h (0–100 *μ*g/mL) [16]. Another group reported that MWCNTs were cytotoxic and cause oxidative stress in dose dependent manner [17, 18].

Several groups suggested that inhalation is the primary route of exposure of MWCNTs and hence induces pulmonary toxicity [19–22]. Recently it was observed that instillation of MWCNTs via oropharyngeal exposure route impairs pulmonary functioning by inducing epithelial damage in C56B1/6 mice due to the development of IL-33 dependent Th-2 associated inflammatory response [23]. Another inhalation study reported that C57BL/6J mice exposed to MWCNTs aerosol (10 mg/m^3^, 5 h/day) for 2, 4, 8, or 12 days showed pulmonary inflammation and damage with rapid development of pulmonary fibrosis in time dependent manner [24].

Therefore, keeping in view the above mentioned reports regarding pulmonary toxicity, present study was designed to examine the toxic effect of bucky tubes [BTs, a type of multiwall carbon nanotubes] on human lung alveolar cell lines (A549).

## 2. Materials and Methods

### 2.1. Chemicals

Bucky tubes (CAS number 308068-56-6), propidium iodide (PI), 2,7-dichlorofluorescein diacetate (DCFDA) dye, 5,5′,6,6′-tetrachloro-1,1′3′3′-tetraethylbenzimidazolecarbocyanine iodide (JC-1) dye, ethidium bromide (EtBr), Triton X-100, 5,5′-dithiobisnitrobenzoic acid (DTNB), and glutathione (GSH) were purchased from Sigma Chemical Co. Ltd. (St. Louis, MO, USA). Phosphate buffered saline (Ca^+2^, Mg^+2^ free; PBS), Dulbecco's modified eagle medium : nutrient mixture F-12 (Ham) (1 : 1) powder (DMEM F-12), trypsin-EDTA, fetal bovine serum (FBS), trypan blue, antibiotic, and antimycotic solution (10,000 U/mL penicillin, 10 mg/mL streptomycin, and 25 *μ*g/mL amphotericin-B) were purchased from Life Technologies Pvt. Ltd. (New Delhi, India). All other chemicals were obtained locally and were of analytical reagent grade. All cell culture plastic wares were obtained from Thermo Scientific Nunc (Rochester, New York).

### 2.2. Characterization of BTs

The particles were characterized by transmission electron microscopy (TEM), dynamic light scattering (DLS), Fourier transform spectroscopy (FTIR), and X-ray diffraction (XRD).

#### 2.2.1. Transmission Electron Microscopy (TEM)

Electron microscopy was carried out for the assessment of morphology and size of BTs. Samples were prepared by suspending BTs in Milli-Q water at a concentration of 25 *μ*g/mL and a drop of nanoparticle suspension was put on the formvar coated copper grids. Then, grids were dried properly and examined under the TEM at an accelerating voltage of 80 kV on TechnaiG2 spirit instrument (FEI, The Netherlands).

#### 2.2.2. Dynamic Light Scattering (DLS)

Bucky tubes were suspended in complete growth medium, that is, DMEMF-12 supplemented with 10% fetal bovine serum (FBS) at a concentration of 150 *μ*g/mL, and probe sonicated (Sonics & Material Inc., New Town, CT, USA) at 30 watt for total 10 min (2.5 min pulse on and 1 min pulse off) and allowed to cool down at room temperature. The average hydrodynamic diameter and zeta potential of BTs were determined using dynamic light scattering and phase analysis light scattering, respectively, using a Zetasizer Nano-ZS, Model ZEN3600 (Malvern Instruments Ltd., Malvern, UK).

#### 2.2.3. Fourier Transform Spectroscopy (FTIR) Analysis of BTs

FTIR analysis of BTs has been performed with the scan range 400–4000 cm^−1^ at the resolution of 8 cm^−1^ using ATR accessory on Agilent Cary 630 FTIR spectrometer.

#### 2.2.4. X-Ray Diffraction (XRD) Analysis of BTs

Powder X-ray diffraction of BTs has been done using Rigaku Miniflex-II bench top X-ray diffractometer with tube voltage of 30 KV.

### 2.3. *In Vitro* Toxicity Assessment

#### 2.3.1. Cell Culture and BTs Exposure

The human lung epithelial cells (A549) were purchased from the National Centre for Cell Sciences (NCCS), Pune, India, and maintained in DMEMF-12 (1 : 1) medium supplemented with 10% heat inactivated FBS, 0.2% sodium bicarbonate, and 1% antibiotic and antimycotic solution at 37°C under a humidified atmosphere of 5% CO_2_.

A549 cells were cultured in complete medium having all supplements and were harvested at 80–85% confluency using 0.25% trypsin-EDTA solution and were seeded at a density of 1 × 10^4^ cells/mL/well in a flat bottom 96-well plate, 1 × 10^5^ cells/mL/well in a 12-well plate, and 2 × 10^5^ cells/mL in a 6-well plate and culture flasks according to the need of the experiment. After 22 h of seeding, cells were incubated with varying concentrations of BTs (1, 10, 25, 50, and 100 *μ*g/mL) for different time points (1 h, 3 h, 6 h, and 24 h) at 37°C; cells without nanoparticles were taken as control.

#### 2.3.2. Intracellular Internalization of Carbon Nanoparticles

The uptake of BTs using flow cytometry was measured according to the method of Suzuki et al. [25]. For assessing intracellular internalization of BTs, cells were seeded in 12-well cell culture plates and after 22 h of seeding, cells were exposed to different concentrations of BTs (1, 10, 25, 50, and 100 *μ*g/mL) for 6 h and 24 h. After completion of exposure time, the culture medium containing nanoparticles was removed and cells were harvested using 0.25% trypsin-EDTA. The cells were then centrifuged at 1200 rpm for 10 min and the pellet was resuspended in 0.5 mL of 1x PBS. The internalization of BTs was measured by flow cytometer (FACS Canto II, BD Biosciences, San Jose, CA, USA) using FACS Diva software (version 6.1.2, BD Biosciences) equipped with a 488 nm laser. Results were expressed as increase in SSC mean as compared to control.

#### 2.3.3. Cytotoxicity Assays

Cytotoxicity of BTs was determined by trypan blue dye exclusion assay and propidium iodide (PI) staining assay.

*Trypan Blue Dye Exclusion Assay.* Viability of A549 cells exposed to BTs was determined by trypan blue dye exclusion assay according to the method of Phillips [26]. In brief, cells were seeded in 24-well cell culture plates and after 22 h of seeding, cells were exposed to different concentrations of BTs for 6 h and 24 h. After completion of exposure time, cells were harvested and centrifuged at 1200 rpm for 10 min. The cell pellet was washed with 1x PBS twice and finally the pellet was resuspended in 200 *μ*L of 1x PBS. Then, 10 *μ*L of sample was gently mixed with 10 *μ*L of trypan blue in an Eppendorf tube and incubated for 5 min at room temperature. This sample was loaded in a chamber slide and counted by using the cell counter (Countess Automated Cell Counter, Invitrogen, UK). The results were expressed as % dead cells when compared with control.

*Propidium Iodide (PI) Staining Assay*. Propidium iodide (PI) dye was used for the flow cytometric assessment of live/dead cells. Briefly, cells were seeded in 12-well cell culture plates and exposed to different concentrations of BTs for 6 h and 24 h. After completion of exposure, cells were harvested using 0.25% trypsin-EDTA and centrifuged at 1200 rpm for 10 min. Supernatant was discarded and the pellet was resuspended in 0.2 mL of 1x PBS containing PI having final concentration of 20 *μ*g/mL and incubated for 10–15 min at 4°C. After incubation, 0.2 mL of 1x PBS was again added and the samples were ready for acquisition using flow cytometer (FACS CantoII, BD Biosciences, San Jose, CA, USA) equipped with a 488 nm laser. Results were analysed using FACS Diva software (version 6.1.2, BD Biosciences) and expressed as percentage cell death when compared with control.

#### 2.3.4. Oxidative Stress Parameters

*(1) Measurement of Intracellular Reactive Oxygen Species (ROS).* The level of intracellular ROS generation was estimated by the method of Wan et al. [27] and modified by Wilson et al. [28] using 2,7-dichlorofluorescein diacetate (DCFDA) dye. Cells were seeded in a 96-well black bottom plate and exposed to different concentrations of BTs for 1 h, 3 h, 6 h, and 24 h and cells without NPs were used as control and a set of experiments without cells were conducted in parallel. Following exposure, the cells were washed twice with 1x PBS and incubated with 20 *μ*M DCFDA dye prepared in 1x PBS for 30 min at 37°C. After completion of incubation time, the reaction mixture was then replaced by 200 *μ*L of PBS and fluorescence intensity was measured in a SYNERGY-HT multiwell plate reader, Bio-Tek (Winooski, USA), using KC4 software at excitation and emission wavelengths of 485 nm and 528 nm, respectively, and results were expressed as percentage ROS generation as compared to control.

*(2) Oxidative Stress Markers*. Cells were cultured in T-75 cm^2^ culture flasks at a final density of ~6 × 10^6^ and exposed for 3 h and 6 h. After exposure, the cells were washed twice with chilled 1x PBS and then scrapped on ice using 1x PBS. The cells were centrifuged at 1200 rpm for 10 min and the pellet was resuspended in 1xPBS to obtain cell lysate. Protein content was measured by Bradford method [29] using BSA as standard.

*Intracellular Glutathione (GSH) Estimation*. GSH content was measured in lysate according to the method of Ellman [30] and expressed as *μ*mole/mg of protein.

*Lipid Peroxidation (LPO) Assay*. The rate of LPO was determined according to the method of Utley et al. [31] by estimating malondialdehyde (MDA) formed with 2-thiobarbituric acid (TBA).

#### 2.3.5. Mitochondrial Membrane Potential (MMP) Analysis Using Lipophilic Fluorochrome

MMP was determined by using fluorescent, lipophilic cationic carbocyanine 5,5′,6,6′-tetrachloro-1,1′3′3′-tetraethylbenzimidazolecarbocyanine iodide (JC-1) dye which exhibits dual fluorescence emission depending upon the membrane potential state of mitochondria. Exposed cells were harvested and washed with 1x PBS and then incubated with 10 *μ*M JC-1 for 15 min at 37°C. After completion of incubation time, the stained cells were diluted with 1x PBS and were assayed using flow cytometer. The red and green fluorescence intensity was measured at an excitation wavelength of 485 nm and an emission wavelength of 520 nm and 600 nm for green and red fluorescence, respectively, using flow cytometry (FACS CantoII, BD Biosciences, San Jose, CA, USA) and results were analysed by using FACS Diva software (version 6.1.2, BD Biosciences).

#### 2.3.6. Apoptosis (Annexin V/PI Double Staining)

Apoptosis on exposure of BTs was measured by using Annexin V-FITC apoptosis detection kit (BD Biosciences, San Jose, CA, USA) as per the manufacturer's protocol. Briefly, treated cells were harvested; cell pellet was washed with 1x PBS and resuspended in 100 *μ*L of 1x binding buffer (10 mM HEPES/NaOH, pH 7.5 containing 140 mM NaCl and 2.5 mM CaCl_2_). 2 *μ*L of Annexin V-FITC and 2 *μ*L of PI were added to 100 *μ*L of cell suspension and incubated for 10 min in dark at 4°C. After completion of incubation time, 400 *μ*L of 1x binding buffer was added and the cells were immediately analyzed by flow cytometry (FACS Canto II, BD Biosciences, San Jose, CA, USA) and results were analyzed by FACSDiva software, version 6.1.2 (BD Biosciences). The results were expressed as FITC negative and PI negative (viable normal cells), FITC positive and PI negative (early apoptotic), FITC positive and PI positive (late apoptotic), and FITC negative and PI positive (necrotic).

#### 2.3.7. Assessment of Chromosomal Damage

Flow cytometric analysis of micronucleus formation (MN) was done according to the method of Pandey et al. [32]. Cells were seeded in 6-well culture plates and exposed for 3 h and 6 h. After the completion of exposure time, treatment was removed, cells were washed with incomplete medium, and fresh growth medium supplemented with 10% FBS was added to each well and incubated till next division cycle. After incubation, cells were harvested and the pellet was resuspended in solution I (containing 10 mM NaCl, 3.4 mM sodium citrate, 10 mg/L RNAse, 0.3 mg/L igepal, 25 mg/L EtBr) and incubated for 45 min at room temperature in dark. Equal volume of solution II (1.5% citric acid, 0.25 M sucrose, 40 mg/L EtBr) was added with vortexing for 2-3 seconds and samples were placed at 4°C, ready for acquisition using flow cytometer (FACS CantoII, BD Biosciences, San Jose, CA, USA) equipped with 488 nm laser and analysed by FACS Diva software (version 6.1.2, BD Biosciences). Results were expressed as percentage micronucleus formed per nucleus.

#### 2.3.8. Assessment of Cell Cycle Progression

Effect of BTs on cell cycle progression was determined by using flow cytometer. Briefly, the treated cells were harvested and fixed in 70% ethanol for 30 min at 4°C and then lysed with 0.2% triton X-100 for 30 min at 4°C. After lysis, cells were incubated for 30 min in dark at 37°C in RNaseA (10 mg/mL) and finally stained with PI (1 mg/mL) for 60 min at 4°C. Then, the flow cytometric analysis was performed using BD FACS Canto II flow cytometer equipped with FACS Diva software (version 6.1.2, BD Biosciences) and results were expressed in percentage cell population in different phases of cell cycle.

### 2.4. Statistical Analysis

All assays were done in three independent sets of experiments and results were expressed as mean ± SEM. Data of treated cells were compared with their respective controls and were analysed using one way analysis of variance (ANOVA) with Dunnett post hoc test to determine significance. In all cases *P* < 0.05 was considered statistically significant.

## 3. Results

### 3.1. Characterisation of Bucky Tubes

**Figure 1:**
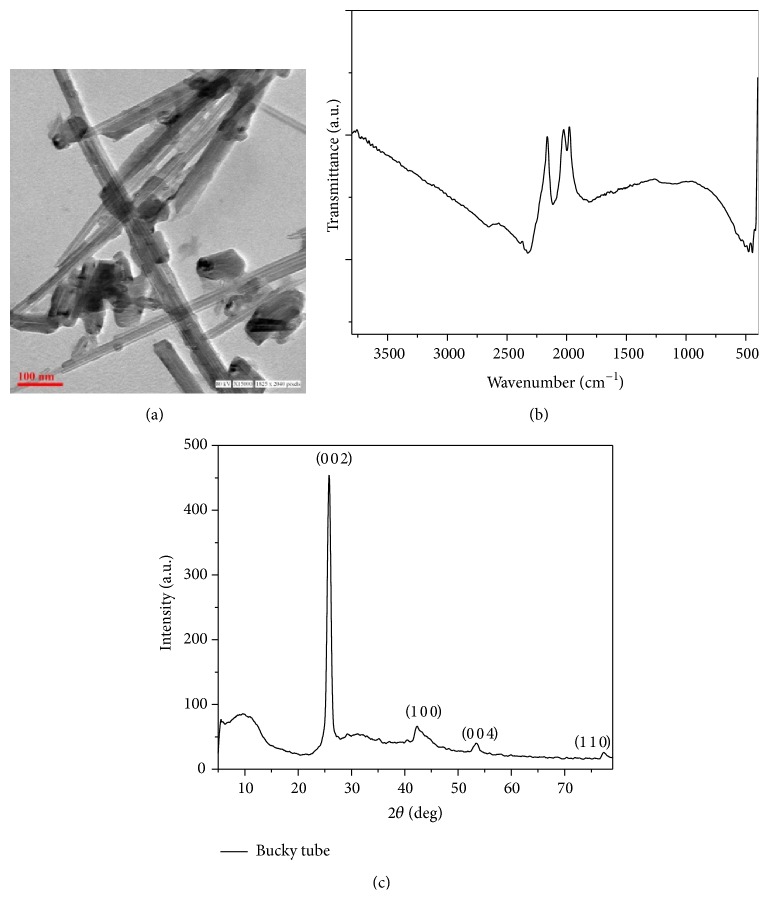
Characterization of BTs: (a) TEM photomicrograph: TEM analysis revealed that BTs were tubular/rod shaped having ~37.2 nm average (bundle) size at scale bar of 100 nm. (b) FTIR analysis revealed the presence of characteristic peaks corresponding to two-dimensional carbon nanostructures. (c) XRD pattern of BTs.

BTs were first analysed by TEM to assess the particle morphology and size. TEM analysis shows that the particles were tubular in shape with an average (bundle diameter) size of ~37.2 nm at scale bar of 100 nm (Figure 1(a)).

Further, the mean hydrodynamic diameter and zeta potential of BTs in cell culture medium DMEM F-12 supplemented with 10% FBS were estimated using DLS and found to be in range of 179.7 nm and 12.9 mV, respectively, with polydispersity index (PdI) 0.354 (Table 1).

FT-IR spectra of BTs exhibited the presence of characteristic peaks at ~2600 cm^−1^, 2325 cm^−1^, 1111 cm^−1^, and 465 cm^−1^ corresponding to the two-dimensional carbon nanostructures (Figure 1(b)).

X-ray diffraction pattern of BTs taken in the 2*θ* range of 5–80° revealed the major diffraction peaks at 25.6°, 42.4°, 53.5°, and 77.2°, which are discussed in detail later (Figure 1(c)).

### 3.2. Intracellular Internalization of Carbon Nanoparticles

**Figure 2:**
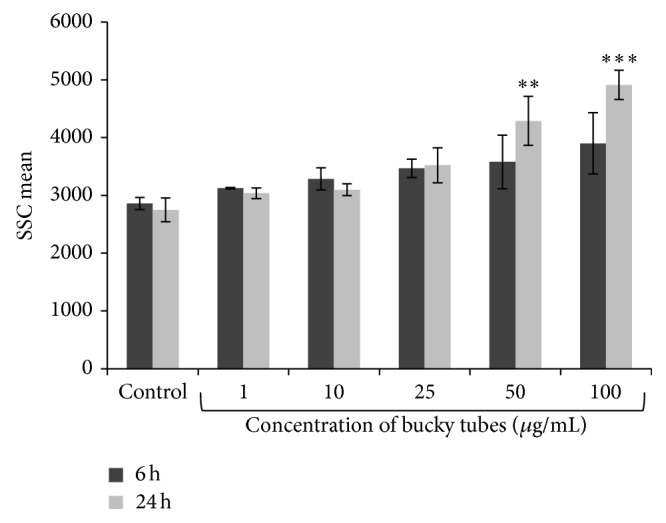
Internalization of BTs in A549 cells after 6 h and 24 h exposure. Increase in SSC mean correlates with the increased granularity of cells which was used as a marker for internalization of NPs. Values represent mean ± SEM of three independent experiments. (^∗∗^ *P* < 0.01, ^∗∗∗^ *P* < 0.001 when compared to control).

The cellular uptake of BTs was assessed by flow cytometry. The change in side scattering (SSC) mean and forward scattering (FSC) mean represents the relative change in granularity and size of the cell, respectively. There was an increase in SSC mean with the increase in concentration at 1, 10, 25, 50, and 100 *μ*g/mL after 6 h and 24 h exposure as represented in Figure 2. The percentage increase in SSC mean of cells treated with BTs was statistically significant (*P* < 0.01, *P* < 0.001) at 50 *μ*g/mL and 100 *μ*g/mL after 24 h exposure.

### 3.3. Cytotoxicity Assessment

**Figure 3:**
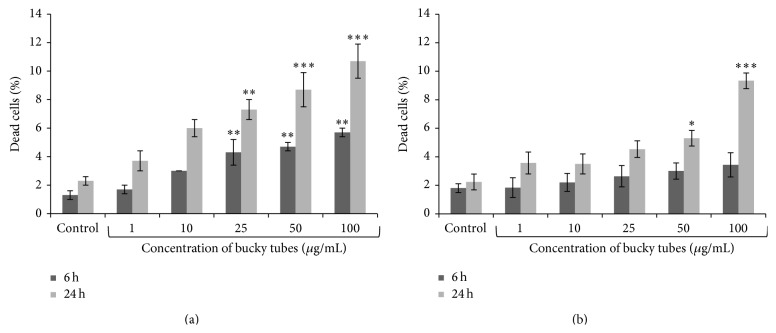
Cytotoxic effect of BTs NPs in A549 cells by (a) trypan blue dye exclusion assay using automatic cell counter and (b) propidium iodide (PI) uptake method in which cells were stained with PI and analysed by flow cytometer after 6 h and 24 h exposure. Results were expressed as the percentage cell death after exposure of BT relative to control cells and were represented as mean ± SEM of three independent experiments. (^∗^ *P* < 0.05, ^∗∗^ *P* < 0.01, and ^∗∗∗^ *P* < 0.001 when compared to control).

For determining cytotoxicity by trypan blue dye exclusion assay and PI staining assay, cells were exposed to varying concentration (1 *μ*g/mL–100 *μ*g/mL) of BTs for 6 h and 24 h. It was revealed from both assays that there was a concentration and time dependent increase in cell death as compared to control. There was statistically significant (*P* < 0.05, *P* < 0.001) increase in cell death from 1.30% to 4.70% and 5.70% after 6 h exposure which increased from 2.30% to 8.70% and 10.7% after 24 h exposure at 50 *μ*g/mL and 100 *μ*g/mL concentrations, respectively, as observed from trypan blue dye exclusion assay (Figure 3(a)). PI staining method showed the similar trend, that is, 3.00%, 3.43% at 6 h and 5.30%, 9.33% at 24 h exposure at 50 *μ*g/mL and 100 *μ*g/mL concentrations, respectively, when compared with respective controls (1.80% and 2.23%) (Figure 3(b)).

### 3.4. Analysis of Oxidative Stress Parameters (Intracellular ROS, GSH, and LPO)

**Figure 4:**
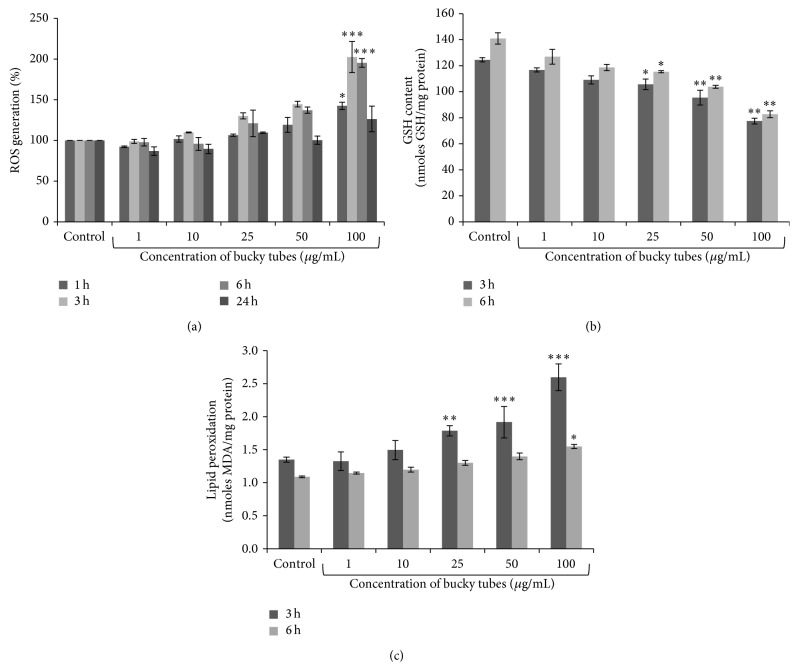
Effect of BTs NPs on (a) induction of intracellular reactive oxygen species (ROS), (b) cellular level of glutathione (GSH), and (c) lipid peroxidation (LPO) in A549 cells. The % ROS generation of the control cells was considered 100% and fluorescence intensity was measured by microplate reader while glutathione level and lipid peroxidation were measured spectrophotometrically. Data represents mean ± SEM of three independent experiments. (^∗^ *P* < 0.05, ^∗∗^ *P* < 0.01, and ^∗∗∗^ *P* < 0.001 when compared to control).

A549 cells were loaded with 2′,7′-dichlorodihydrofluorescein diacetate and it was observed that BTs induced ROS generation in both time and concentration dependent manner which was revealed from increase in DCF fluorescence. The fluorescence intensity increased from 101.71%, 106.37%, 119.21%, and 142.49% after 1 h and 109.77%, 130.12%, 144.55%, and 202.43% after 3 h exposure of BTs as compared to control. The fluorescence intensity was decreased from 95.7%, 120.98%, 137.11%, and 195.23% after 6 h and 89.58%, 109.57%, 100.29%, and 126.32% after 24 h exposure of BTs at concentrations (10–100 *μ*g/mL) as compared to control (Figure 4(a)). A statistically significant reduction (*P* < 0.05, *P* < 0.001) in cellular GSH content (Figure 4(b)) with increase in lipid peroxidation (Figure 4(c)) was observed after 3 h and 6 h exposure to BTs in A549 when compared with respective control.

### 3.5. Detection of Changes in Mitochondrial Membrane Potential

**Figure 5:**
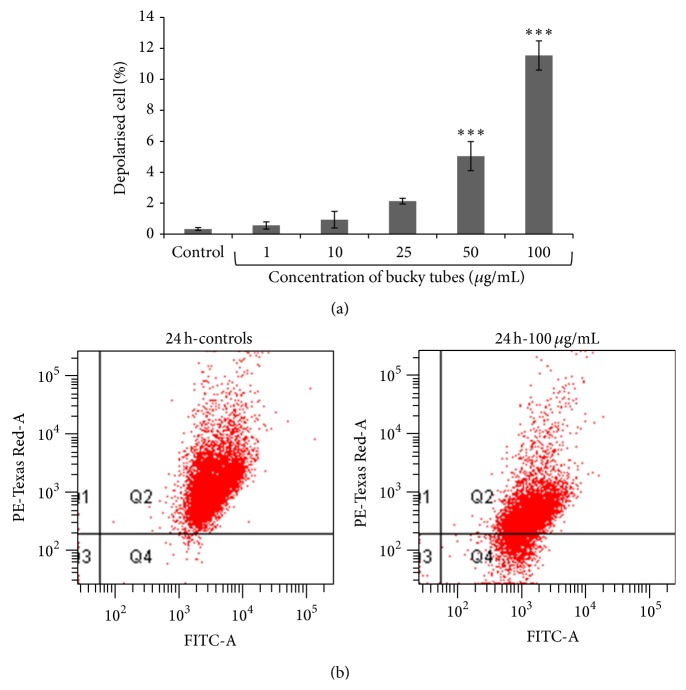
Flow cytometric detection of change in mitochondrial membrane potential (MMP) in A549 cells exposed to BTs using JC-1 dye. (a) Representative bar graph of three independent experiments analysed. (b) Representative dot plots. Data represent mean ± SEM of three experiments. (^∗∗∗^ *P* < 0.001 when compared to control).

Cells treated with BTs showed a statistically significant (*P* < 0.001) mitochondrial membrane depolarization which was detected by JC-1 dye using flow cytometer. Normal healthy (polarized) cells showed higher levels of fluorescence emission measured in red channel while cells in which dye does not accumulate in mitochondria showed depolarized membrane having more fluorescence in green channel. Our data represents 5.03% and 11.53% depolarized cells at 50 *μ*g/mL and 100 *μ*g/mL when compared with control cells (0.33%) (Figures 5(a) and 5(b)).

### 3.6. Apoptosis (Annexin V/PI Double Staining)

**Figure 6:**
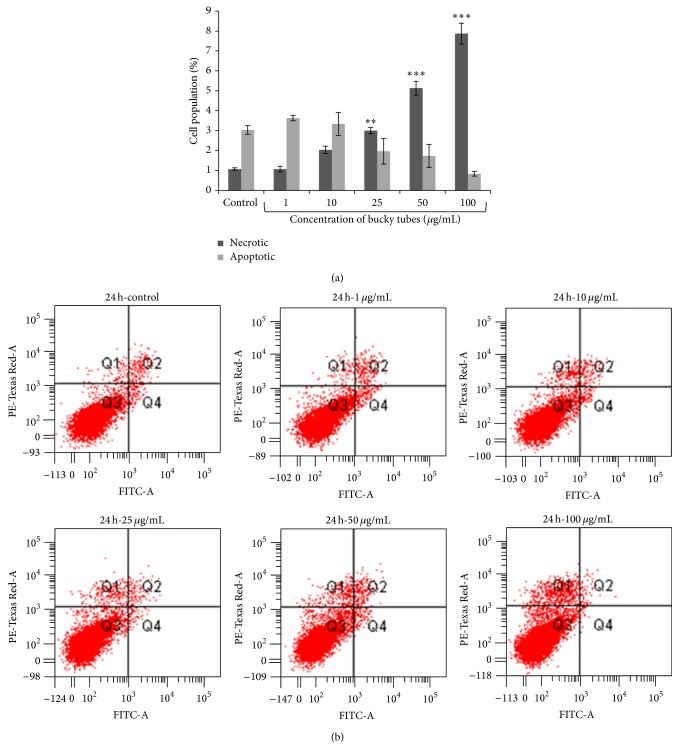
BTs induced cell death in A549 cells analysed by flow cytometry. Data represent mean ± SEM of three experiments. (a) Representative bar graph. (b) Dot plots. (^∗∗^ *P* < 0.01, ^∗∗∗^ *P* < 0.001 when compared to control).

The cell population of interest was gated on the basis of untreated control stained cells. These were divided into four quadrants and were analysed as FITC negative and PI negative (viable normal cells), FITC positive and PI negative (early apoptotic), FITC positive and PI positive (late apoptotic), and FITC negative and PI positive (necrotic). It was observed that there was increase in percentage of FITC^−^ and PI^+^ population and decrease in FITC^+^ PI^+^ and FITC^+^ PI^−^ population with increasing concentration which suggest that at lower concentration BTs cause apoptosis in A549 cells and at higher concentration cause necrosis (Figures 6(a) and 6(b)).

### 3.7. Chromosomal Damage and Progression of Cell Cycle

**Figure 7:**
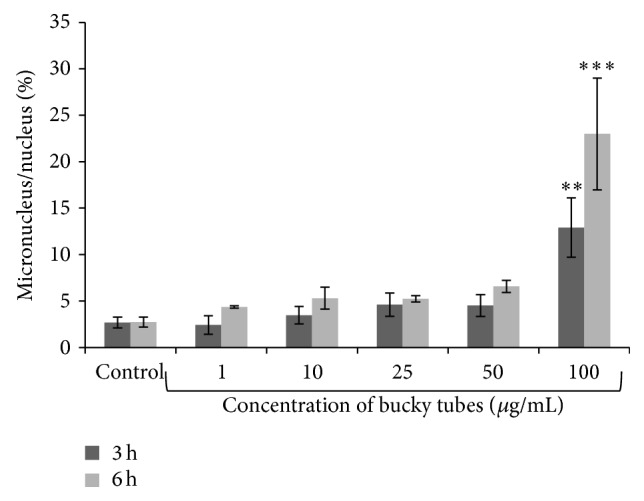
Flow cytometric detection of chromosomal damage by micronucleus formation after 3 h and 6 h of exposure. Data represent mean ± SEM of three experiments. (^∗∗^ *P* < 0.01, ^∗∗∗^ *P* < 0.001 when compared to control).

A statistically significant (*P* < 0.05) induction of MN formation was observed in A549 after 3 h and 6 h exposure to BTs from 12.9% and 23.0%, respectively, at 100 *μ*g/mL by flow MN with respect to control 2.7% (Figure 7).

**Figure 8:**
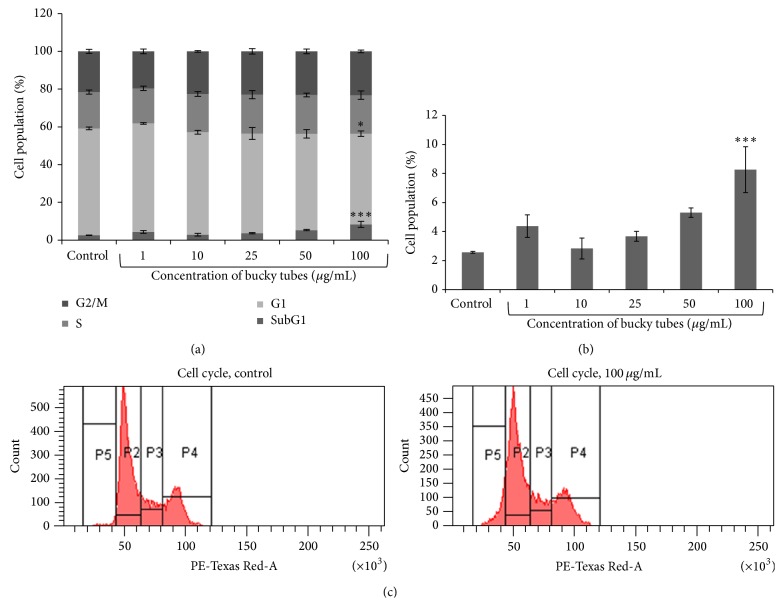
Flow cytometric analysis of cell cycle of A549 cells exposed to BTs for 24 h. (a) Bar graph representing cell population in different phases of cell cycle and (b) representing % cell population in sub-G1 phase which ultimately represents apoptotic population and (c) representative histograms. (^∗^ *P* < 0.05, ^∗∗∗^ *P* < 0.001 when compared to control).

Flow cytometric analysis of cell cycle of A549 cells exposed to BTs for 24 h revealed that there was a significant concentration dependent increase in cells present in sub-G1 phase (4.4%, 2.8%, 3.7%, 5.3%, and 8.3%) in comparison to control (2.6%) while there is no significant change in percentage of cells in G1, S, and G2/M phase of cell cycle which revealed that there was increase in apoptotic cell population (Figures 8(a), 8(b), and 8(c)).

## 4. Discussion

MWCNTs are produced in huge amount and exponentially applied in almost all sectors. These increasing applications necessitate the evaluation of risk of MWCNTs exposure and their adverse health effects.

Therefore, present study was designed to evaluate the toxicity of BTs (type of MWCNTs) and add to the present knowledge by concluding that BTs get internalized and cause cellular toxicity. It causes oxidative stress by generation of intracellular ROS which induce lipid peroxidation that leads to imbalance in level of antioxidants. It has been also observed that BTs exposure causes mitochondrial dysfunctioning, chromosomal damage, and cell cycle arrest for which oxidative stress may be one of the possible reasons.

Prior to investigating the* in vitro* toxicity, characterization of experimental nanoparticles is essential as the shape, size, particle dispersity, and charge on its surface primarily affect the biological responses [33]. Hence, first of all we examined BTs by electron microscopy (TEM) which is the most widely used technique and directly measures the particle size and morphology. Size of particles observed by TEM was ~37.2 nm (bundle diameter) having tubular structure of the diameter ranging from 5 to 10 nm.

Another method used was DLS which characterize the particle in the cell culture medium which was used for treatment of BTs to A549 so as to characterise the particle by simulating the culture conditions. The mean hydrodynamic diameter and zeta potential of BTs in culture medium obtained from DLS were in the range of 179.7 nm and 12.9 mV, respectively, at physiological pH which was more than the size reported (7–12 nm) by its commercial supplier (Sigma-Aldrich). The reason behind the vast difference in particle size may be that DLS characterise the particle considering it spherical in shape which is not in the case of BTs as these are tubular in structure. Moreover, difference in particle size may be due to agglomeration which is influenced by intrinsic and extrinsic factors [33].

A FT-IR spectrum of BTs illustrates the presence of characteristic peaks related to 2-dimensional carbon nanostructures. Stretching at around ~2600 cm^−1^ has been assigned to C–O stretching that may correspond to the presence of –COOH groups. Several minor peaks appeared due to amorphous content, C–H stretching vibrations (~2325 cm^−1^, ~1111 cm^−1^, and ~465 cm^−1^), and so forth; stretching at ~2100 cm^−1^ is due to the interference of CO_2_ during the course of the spectral measurements.

X-ray diffraction pattern of BTs showed the graphitic structure with interlayer spacing of 0.33 nm corresponding to the d_002_ reflection at 25.6°. Peaks at 42.4°, 53.5°, and 77.2° were attributed to the diffraction of (1 0 0), (0 0 4), and (1 1 0) planes, respectively, which suggested the BTs structural resemblance to the multiwalled carbon nanotubes.

To explore the toxicity potential of BTs, it is necessary to evaluate the internalization of BTs which can be correlated with varying biological responses. The cellular uptake of nanoparticles was considered as a primary method of screening of nanotoxicity by flow cytometer [25] and therefore, BTs were examined by measuring the relative change in cell granularity (SSC). Our preliminary study on A549 cells demonstrated a significant increase in SSC mean (marker of granularity) in a time and concentration dependent manner which suggests that there was uptake of BTs in treated cells when compared with untreated cells. There were several reports which suggest that increase in SSC mean is the indicator of increase in cellular granularity on the internalization of NPs [34–36].

After internalization studies, effect of BTs on cellular viability was examined by trypan blue dye exclusion assay and PI assay. It was found that BTs exposure causes a statistically significant decrease in cell viability in lung alveolar cells in both assays at higher concentration at different time points. Several groups have reported that, after internalization, NPs interact with cellular components that induce alteration in cellular/functional responses which ultimately leads to cell death [34, 37].

Further, to investigate the cause of cytotoxicity, we tried to identify the generation of free radicals which are considered to be the primary cause of toxicity due to exposure of NPs [35, 38–40]. On analysing the effects of BTs exposure to A549 at different time points, we found that these induced intracellular ROS generation up to 3 h and decreased further. This decrease in ROS after 3 h may be due to the decrease in viable cells or ROS generated may be stabilized after certain duration.

It is documented that oxidative stress occurs in a cell or tissue when the concentration of ROS generated exceeds the antioxidant capability of that cell [41–43]. Therefore, we tried to determine the level of one of the major endogenous antioxidants and free radical scavengers, that is, glutathione, which is mostly (90%) present in reduced form in healthy cells and in the present study, there was decrease in level of GSH in treated cells as compared to untreated one which may be possibly due to elevated level of intracellular free radicals.

In addition, overproduction of ROS may result in damage to critical biomolecules including cellular fatty acids which are readily oxidized to produce lipid peroxyl radicals and lipid hydroperoxides. Lipid peroxyl radicals are subsequently propagated into malondialdehyde (MDA) which is the major carbonyl produced during LPO and potent mutagen and carcinogenic compound. We explored the level of cellular fatty acids and found that there was increase in level of LPO with increasing concentration which represents the increased toxicity due to free radical generation.

Based on a study on wide body of literature it is seen that severe oxidative stress can cause instability in mitochondrial membrane potential, DNA damage, and/or cell death and even moderate oxidation can trigger apoptosis, while more intense stresses may cause necrosis [44, 45]. In the present study, MMP was evaluated by JC-1 dye which is positively charged, having dual fluorescence spectra and gets accumulated in the electronegative leaflet (interior) of polarised mitochondria as aggregates. Change in redox potential causes depolarization of mitochondrial membrane allowing mitochondrial permeability transition pores to pass JC-1 aggregates from interior to outer environment and reside there as monomers. Our results indicated that there was a statistically significant increase in JC-1 monomers at 50 *μ*g/mL and 100 *μ*g/mL which was correlated with the loss in mitochondrial membrane potential. Our results were in accordance with previous reports which showed that the exposure of nanoparticles causes toxicity and alteration in mitochondrial functioning which may be due to loss in membrane potential [37, 46, 47].

Moreover, it can be said that disruption of mitochondrial activity is the distinctive feature in cell death and in accordance with this we assessed cell fate by Annexin V/PI double staining and found that at lower concentration BTs cause apoptosis in A549 cells and at higher concentration causes necrosis. It can be concluded that BTs were mild toxic towards A549 and lead to cell death. Some of the previous reports have shown that mitochondrial dysfunction is activated with accidental cell death (necrosis) or programmed cell death [48, 49].

Another important outcome of these deleterious reactive radicals is that they can diffuse through membranes and may also interact with cellular DNA and nitrogen bases by forming adducts resulting in a loss of cellular homeostasis [50]. It causes chromosomal damage through micronucleus formation which arises when acentric chromosomal fragments and/or whole chromosome are excluded from the main nucleus at the telophase of cell division. Hence, micronucleus assay was used for detecting the genotoxic potential of those agents that induce chromosomal breakage and instability in spindle formation [51]. We evaluated the genotoxic potential (micronucleus formation) of BTs in A549 by flow cytometric method and observed that BTs exposure causes the increase in number of MN formed with the increase in concentration as analysed by flow cytometer suggesting chromosomal damage.

Further, in response to DNA damage, progression of cell cycle was assessed by PI staining flow cytometrically and it was observed that there was statistically significant increase in apoptotic cell population in exposed cells at 100 *μ*g/mL (indicated by Sub-G1 phase) as compared to control.

## 5. Conclusion

Present study demonstrated that BTs get internalized intracellularly to human lung alveolar cells and cause cell death with increasing concentrations and time points which was observed by flow cytometer. Exposure of these nanoparticles causes increased ROS production and lipid peroxidation with concomitant depletion in glutathione level which confirms the induction of oxidative stress which ultimately triggers loss in MMP at 100 *μ*g/mL concentration. Along with this, BTs cause oxidative stress which may lead to chromosomal damage causing micronucleus formation and cell death as revealed from Annexin V/PI double staining and cell cycle analysis. Overall study determines that these BT NPs were mild toxic towards human lung alveolar cell lines showing statistically significance but seems to be less significant biologically. So, these CNMs can be considered to be safe in some specific cases.

## Figures and Tables

**Table 1:** Characterisation of BTs by DLS.

S. Number	Medium	Hydrodynamic diameter	PdI	Zeta potential
1	Culture medium (DMEM F-12 supplemented with 10% FBS)	179.7 nm	0.354	12.9 mV
